# Limited transmission of V180I genetic Creutzfeldt-Jakob disease in knock-in mice models

**DOI:** 10.1099/jgv.0.002224

**Published:** 2026-03-18

**Authors:** Kenta Teruya, Shirou Mohri, Tetsuyuki Kitamoto

**Affiliations:** 1 Office for Research Initiative and Development, Nagasaki University, 1-14 Bunkyo, Nagasaki, Japan; 2 Department of Neurochemistry, Tohoku University School of Medicine, Seiryo 2-1, Sendai, Japan; 3 Department of Neurological Science, Tohoku University School of Medicine, Seiryo 2-1, Sendai, Japan; 4 Department of Laboratory Medicine, National Center of Neurology and Psychiatry, Kodaira 4-1-1, Tokyo, Japan

**Keywords:** Creutzfeldt-Jakob disease (CJD), human prion, infectivity, knock-in mouse, V180I mutation

## Abstract

Differentiating Creutzfeldt-Jakob disease (CJD) with the V180I mutation from other types of dementia is extremely difficult. Additionally, its differentiation is sometimes determined after performing neurosurgery, which is associated with a high risk of V180I prion contamination; however, the infectivity of the V180I prion has not been properly investigated. Especially in East Asia, this issue must be addressed to respond effectively to accidental contamination that leads to iatrogenic CJD. The results of our transmission experiments involving various humanized knock-in mice clearly indicate that the transmissibility of tissue from V180I genetic CJD cases is significantly limited.

**Impact Statement**

This study provides the first experimental evidence of the transmissibility of V180I genetic Creutzfeldt-Jakob disease. By using various humanized knock-in mice models and performing serial dilutions of MM1 and VV2 prions as reference standards, this study established a comparative framework for assessing the transmission risk of V180I prion. These findings underscore that the V180I prion has a distinct profile; therefore, subtype-specific risk assessments of contamination are necessary.

Since we reported the V180I mutation in human prion protein (PrP) in 1993 [1], it has become the most common pathogenic mutation in genetic Creutzfeldt-Jakob disease (gCJD) in Japan [2]. The V180I mutation is characterized by its late onset and low penetrance [3]; therefore, some individuals with this mutation do not develop Creutzfeldt-Jakob disease (CJD). Additionally, it is often mistakenly diagnosed as an unknown or other type of dementia because patients with gCJD with the V180I mutation (V180I-gCJD) experience slower progression than that associated with sporadic CJD (sCJD) [3,5]. Unfortunately, in some cases, the V180I mutation is evident only after neurosurgery and is considered an incident case (Kitamoto, personal communication). Individuals exposed to incompletely sterilized neurosurgical instruments during an incident case are considered at increased risk for CJD. They are informed of the exposure and asked to avoid potential transmission to others through blood and organ donations; additionally, they are clinically followed up for 10 years.

One case involving pathology similar to that of V180I-gCJD in a cadaver used in anatomical practice has been reported [6]. Therefore, concerns regarding potential exposure to prions during surgical training involving cadavers have been raised [7]. The number of V180I cases in Japan is higher than that in Western countries [3]; therefore, the risk of prion infection attributable to V180I-gCJD may not be considered as often in those countries.

Because it is essential to evaluate the risk of prion infection attributable to V180I-gCJD, we aimed to compare the latent infectivity of prions associated with V180I with the infectivity of MM1-sCJD and VV2-sCJD based on brain weight. Therefore, we performed transmission experiments using brain homogenates (BHs) derived from MM1-sCJD, VV2-sCJD and V180I-gCJD cases. The BHs for V180I-gCJD (H-28, I-62) (Table 1), as well as the sCJD (MM1 and VV2) controls, were prepared from the frontal cortex region using a method similar to that previously described [8]. To ensure consistency, tissues for Western blotting were sampled from regions adjacent to those used for BH preparation. In V180I-gCJD, the cerebral cortex is the region most severely affected by pathological changes [4], making it the primary target for our analysis. Western blot analysis (Fig. 1) revealed that the V180I-gCJD BHs contained a sufficient amount of V180I-prion for the study. Notably, these signals exhibited the hallmark characteristic of V180I prions: the absence of the diglycosylated form of proteinase K-resistant PrP (PrPres).

**Table 1.** Results of transmission experiments involving each inoculum from two V180I-gCJD cases. V180I-gCJD cases (upper portion). Some models did not exhibit successful transmission (lower portion)

	H-28 (surveillance no. 654)	I-62 (surveillance no. 691)
Sex	Female	Male
Age at the time of death	89	73
Illness duration (months)	29	9
Mutation	V180I	V180I
Codon 129	MM	MM
Codon 219	EE	EE
**Inoculated model**	**Attack rate (incubation days)**	**Attack rate (incubation days)**
Ki-129M/M	0/5 (447, 725, 745, 746, 812)	0/4 (769, 812, 812, 812)
Ki-129V/V	0/4 (725, 812, 812, 812)	0/6 (563, 612, 740, 780, 812, 812)
Ki-219K/K	0/4 (504, 539, 740, 752)	0/5 (546, 614, 650, 740, 812)
Ki-ChM	0/3 (457, 742, 742)	0/4 (545, 573, 741, 804)
Ki-bank vole	0/9 (622, 677, 677, 698, 737, 737, 800, 868, 868)	0/8 (463, 463, 483, 582, 632, 651, 665, 691)
Ki-180I/I	0/6 (545, 651, 676, 784, 829, 885)	0/6 (451, 561, 734, 801, 816, 861)

**Fig. 1.**
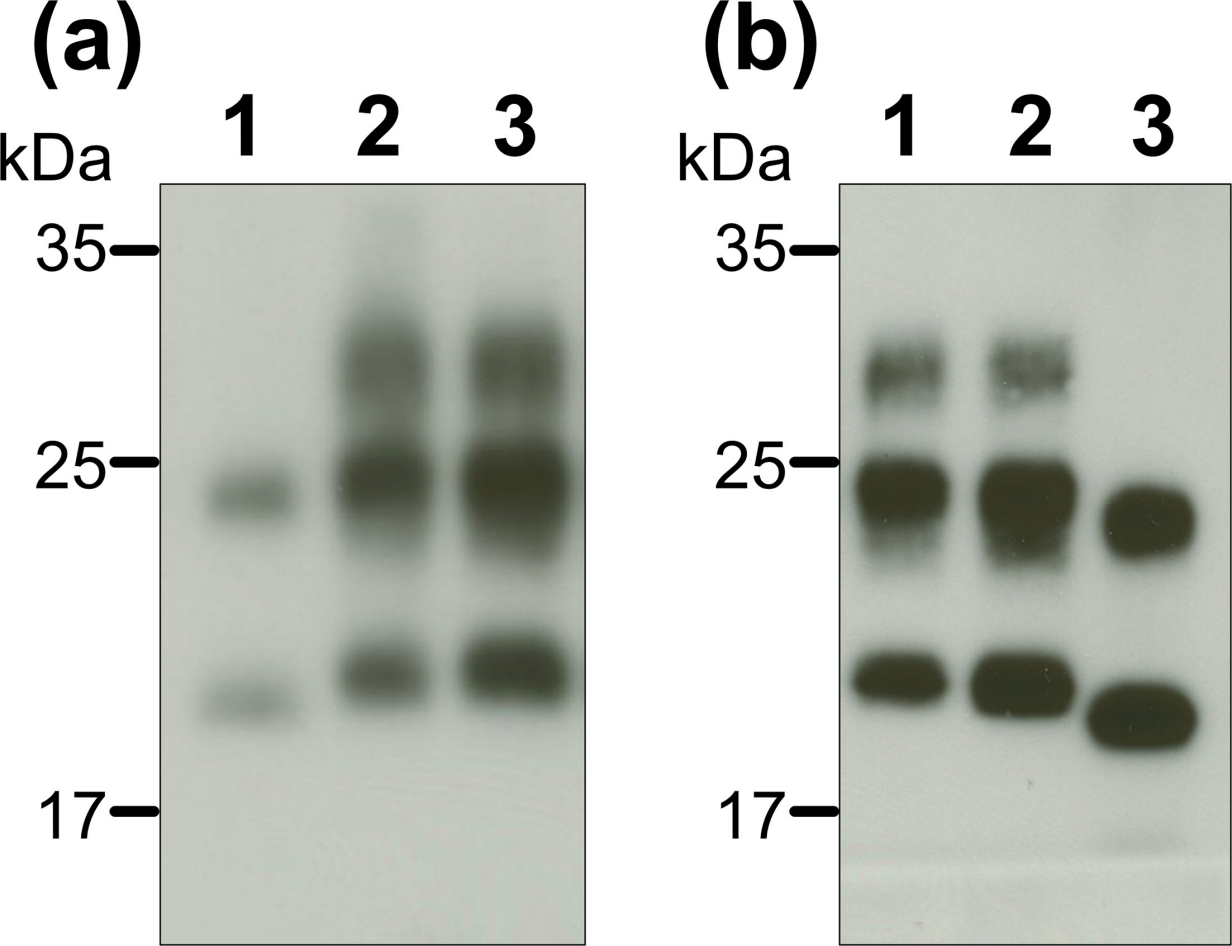
Detection and evaluation of PrPres in BHs. (a) Lane 1: V180I-gCJD (H-28, undiluted), lane 2: MM1-sCJD (fourfold dilution), lane 3: sCJD-MM1 (ninefold dilution). (b) Lane 1: MM1-sCJD (ten-fold dilution), lane 2: MM1-sCJD (threefold dilution), lane 3: V180I-gCJD (I-62, undiluted). Molecular weight markers (kDa) are indicated on the left of each panel. Lanes 2 and 3 in (a), and lanes 1 and 2 in (b), are derived from different MM1-sCJD cases. PrPres were detected using the 3F4-antibody.

We previously conducted transmission experiments involving WT mice [9] that showed a moderate attack rate of 64% (85/132) with BHs (10% w/v) derived from 129MM-sCJD cases, a low attack rate of 24.7% (20/81) with BHs derived from 129MV cases and unsuccessful transmission (0/11) with BHs derived from 129VV cases. WT mice were not suitable for serial dilution studies. However, knock-in mice expressing human PrP genes consistently exhibited 100% transmission of both genotypes [10], thus enabling infectivity assessments using serial dilution of BHs. Accordingly, we conducted transmission experiments using human PrP knock-in mice to assess the degree of infectivity of V180I-gCJD tissue.

During the transmission experiment involving V180I-gCJD prions, 20 µl of BHs (10% w/v, denoted as 10^−1^) was administered intracerebrally. To assess the infectivity of WT *PRNP* backgrounds in humans, we used Ki-129M/M and Ki-129V/V knock-in mice, which are highly susceptible to MM1 and VV2 prions, respectively. For the transmission experiments involving MM1 and VV2, BHs were subjected to serial dilution (10^−1^ to 10^−7^). Chimeric model animals [8], such as Ki-ChM, which are more susceptible to MM1 prions, were not used in the serial dilution study to evaluate infectivity in humans. Mice were observed over time and killed when disease onset was confirmed or when they became weak. One hemisphere of the brain was frozen and subjected to Western blotting, and the other hemisphere, spleen, lymph nodes and small intestines were fixed in formalin and subjected to histological examinations [8].

Transmission experiments involving two V180I mutation cases were performed (Table 1). Both cases were registered in the Japanese CJD surveillance system and exhibited typical genetic, pathological and Western blotting features of V180I-gCJD [11]. No positive findings of haematoxylin and eosin staining for spongiform changes, PrP immunostaining or Western blotting were observed in the Ki-129M/M, Ki-129V/V and Ki-219K/K mice [12], in which human WT PrP was introduced (Table 1). In addition to knock-in mice with the human WT gene, other mice susceptible to distinct prions were examined during our transmission experiments involving V180I. Ki-ChM mice, which are a chimeric type highly sensitive to MM1 prions, Ki-bank voles, which are sensitive to various prions, and Ki-180I/I mice, which possess the protein sequence corresponding to the expected V180I prion, did not exhibit positive findings [8,13]. Ki-180I/I mice were susceptible to the VV2 prion and resistant to the MM1 prion (Kitamoto, personal communication). However, Ki-180I/I mice were not susceptible to V180I-gCJD BHs, which was an unexpected finding (Table 1).

To estimate the relative infectivity of V180I-gCJD BHs in humanized mice, we conducted comparative transmission experiments using serially diluted inocula of MM1 and VV2 BHs. These two prions are commonly detected in both growth hormone treatment-associated CJD [14] and dura mater grafted-associated CJD [15]. In addition to these cases, Kuru is strongly suggested to originate from the transmission of the sCJD VV2 prion strain [16]. Therefore, it is meaningful to compare the infectivity of V180I-gCJD BHs with that of MM1-sCJD or VV2-sCJD BHs. Serially diluted (from 10^−1^ to 10^−7^) MM1 and VV2 BHs from Ki-129M/M or Ki-129V/V mice were prepared, and transmission experiments were performed (Table 2). Even when diluted to 10^−4^, MM1 BHs were transmitted in the Ki-129M/M mice, with an attack rate of 100%. Notably, an attack rate of 100% was observed with VV2 BHs even when further diluted to 10^−5^. A comparison of the Ki-129V/V and Ki-129M/M mice indicated that Ki-129M/M mice exhibited a longer incubation period, which may have contributed to the difference in infectivity between the 10^−4^ and 10^−5^ dilutions. However, we previously conducted transmission experiments involving Ki-ChM mice, which had a shorter MM1 BH incubation period than that of Ki-129M/M mice, and found that dilution to 10^−4^ resulted in an attack rate of 100% [8]. Additionally, MM1 BHs diluted to 10^−5^ and 10^−6^ resulted in attack rates of 40% (attack rate, 2/5) and 20% (1/5), respectively, in Ki-ChM mice [8]. Despite their longer incubation period than that of Ki-ChM mice, Ki-129V/V mice exhibited transmission with VV2 BHs diluted to 10^−6^ (attack rate, 50%; 2/4) and 10^−7^ dilution (attack rate, 20%; 1/5). This finding suggests that compared with MM1 BHs, VV2 BHs remain infectious at higher dilutions.

**Table 2.** Transmission experiments involving serially diluted MM1 and VV2 BHs. The incubation days represent those of all mice in the experiment. Bold and underlined numbers represent confirmed transmissions. Transmission was confirmed by observed symptoms, Western blotting and immunohistochemistry results. sd, standard deviation

Inoculated model	Prion, dilution	Attack rate (incubation days)	Mean±sd
Ki-129M/M	MM1, 10^−1^	**4/4** (**504**, **539**, **546**, **546**)	**534**±**20.1**
	MM1, 10^−2^	**5/5** (**529**, **540**, **539**, **546**, **552**)	**541**±**8.1**
	MM1, 10^−3^	**5/5** (**521**, **529**, **635**, **692**, **707**)	**617**±**88**
	MM1, 10^−4^	**5/5** (**510**, **665**, **692**, **707**, **707**)	**656**±**83.5**
	MM1, 10^−5^	1/5 (500, 574, **712**, 756, 805)	–
	MM1, 10^−6^	0/5 (735, 815, 815, 815, 815)	–
	MM1, 10^−7^	0/5 (500, 634, 727, 809, 815)	–
Ki-129V/V	VV2, 10^−1^	**6/6** (**307**, **307**, **307**, **331**, **331**, **331**)	**319**±**13.1**
	VV2, 10^−2^	**7/7** (**322**, **322**, **322**, **331**, **341**, **341**, **345**)	**332**±**10.3**
	VV2, 10^−3^	**6/6** (**310**, **317**, **322**, **350**, **356**, **356**)	**335**±**21.1**
	VV2, 10^−4^	**6/6** (**350**, **356**, **356**, **373**, **407**, **415**)	**376**±**28.1**
	VV2, 10^−5^	**5/5** (**391**, **428**, **460**, **460**, **464**)	**441**±**31.3**
	VV2, 10^−6^	2/4 (**526**, **622**, 728, 728)	–
	VV2, 10^−7^	1/5 (300, **474**, 642, 728, 728)	–

During the V180I-gCJD BH transmission experiments, Ki-129M/M and Ki-129V/V mice were inoculated with 10% BHs (10^−1^). Collectively, the estimated infectivity of V180I-gCJD BHs was less than 1/10,000 of that of MM1 BHs and less than 1/1,000,000 of that of VV2 BHs. It should be mentioned that the concentration of the proteinase K-resistant form of PrP from V180I-gCJD was lower (1/10–1/30) than that of MM1-sCJD and VV2-sCJD [17]. Because this study aimed to investigate the degree of transmissibility of contamination from V180I BHs, we compared infectivity based on the brain weight of cases with MM1 and VV2. Among humans, the risk of prion infection with V180I-gCJD tissue is predicted to be significantly lower than that with MM1-sCJD and VV2-sCJD. This study was limited by the inability to conduct long-term observations beyond the typical lifespan of mice. Nevertheless, no evidence of V180I-gCJD BH transmissibility was observed under the experimental conditions. Our results are consistent with the low seeding activity *in vitro* of V180I prion, as reported previously [18].

## Abbreviations

BHs: brain homogenates
CJD: Creutzfeldt-Jakob disease
gCJD: genetic Creutzfeldt-Jakob disease
PrP: prion protein
PrPres: proteinase K-resistant prion protein
sCJD: sporadic Creutzfeldt-Jakob disease
V180I-gCJD: genetic Creutzfeldt-Jakob disease with the V180I mutation

## References

[1] Kitamoto T, Ohta M, Doh-ura K, Hitoshi S, Terao Y (1993). Novel missense variants of prion protein in Creutzfeldt-Jakob disease or Gerstmann-Sträussler syndrome. Biochem Biophys Res Commun.

[2] Kai H, Teruya K, Takeuchi A, Nakamura Y, Mizusawa H (2023). Preventive or promotive effects of *PRNP* polymorphic heterozygosity on the onset of prion disease. Heliyon.

[3] Minikel EV, Vallabh SM, Lek M, Estrada K, Samocha KE (2016). Quantifying prion disease penetrance using large population control cohorts. Sci Transl Med.

[4] Jin K, Shiga Y, Shibuya S, Chida K, Sato Y (2004). Clinical features of Creutzfeldt-Jakob disease with V180I mutation. Neurology.

[5] Tomizawa Y, Taniguchi D, Furukawa Y (2020). Genetic Creutzfeldt-Jakob disease mimicking dementia with Lewy bodies: clinical and radiological findings. J Neurol Sci.

[6] Nakagaki T, Kaneko M, Satoh K, Murai K, Saiki K (2022). Detection of prions in a cadaver for anatomical practice. N Engl J Med.

[7] Ogami-Takamura K, Saiki K, Endo D, Murai K, Tsurumoto T (2022). The risk of Creutzfeldt-Jakob disease infection in cadaveric surgical training. Anat Sci Int.

[8] Taguchi Y, Mohri S, Ironside JW, Muramoto T, Kitamoto T (2003). Humanized knock-in mice expressing chimeric prion protein showed varied susceptibility to different human prions. Am J Pathol.

[9] Tateishi J, Kitamoto T, Hoque MZ, Furukawa H (1996). Experimental transmission of Creutzfeldt-Jakob disease and related diseases to rodents. Neurology.

[10] Kobayashi A, Asano M, Mohri S, Kitamoto T (2007). Cross-sequence transmission of sporadic Creutzfeldt-Jakob disease creates a new prion strain. J Biol Chem.

[11] Matsubayashi T, Sanjo N (2022). Systematic review of clinical and pathophysiological features of genetic Creutzfeldt-Jakob disease caused by a val-to-ile mutation at codon 180 in the prion protein gene. Int J Mol Sci.

[12] Hizume M, Kobayashi A, Teruya K, Ohashi H, Ironside JW (2009). Human prion protein (PrP) 219K is converted to PrPSc but shows heterozygous inhibition in variant Creutzfeldt-Jakob disease infection. J Biol Chem.

[13] Kobayashi A, Matsuura Y, Takeuchi A, Yamada M, Miyoshi I (2019). A domain responsible for spontaneous conversion of bank vole prion protein. Brain Pathol.

[14] Ritchie DL, Barria MA, Peden AH, Yull HM, Kirkpatrick J (2017). UK Iatrogenic Creutzfeldt-Jakob disease: investigating human prion transmission across genotypic barriers using human tissue-based and molecular approaches. Acta Neuropathol.

[15] Kobayashi A, Kitamoto T, Mizusawa H (2018). Iatrogenic Creutzfeldt-Jakob disease. Handb Clin Neurol.

[16] Parchi P, Cescatti M, Notari S, Schulz-Schaeffer WJ, Capellari S (2010). Agent strain variation in human prion disease: insights from a molecular and pathological review of the National Institutes of Health series of experimentally transmitted disease. Brain.

[17] Watanabe M, Nakamura K, Saito R, Takeuchi A, Takahashi T (2023). V180I genetic Creutzfeldt-Jakob disease: severe degeneration of the inferior olivary nucleus in an autopsied patient with identification of the M2T prion strain. Neuropathology.

[18] Wang Z, Yuan J, Shen P, Abskharon R, Lang Y (2019). In vitro seeding activity of glycoform-deficient prions from variably protease-sensitive prionopathy and familial CJD associated with PrP^V180I^ mutation. Mol Neurobiol.

